# Factors Associated With Prolonged-Stay Patients Within the Post-anesthesia Care Unit: A Cohort Retrospective Study

**DOI:** 10.7759/cureus.60092

**Published:** 2024-05-11

**Authors:** Leen Alghamdi, Razan Filfilan, Arwa Alghamdi, Roza Alharbi, Haifaa Kayal

**Affiliations:** 1 College of Medicine, King Saud Bin Abdulaziz University for Health Sciences, Jeddah, SAU; 2 Anesthesiology, King Saud Bin Abdulaziz University for Health Sciences, Jeddah, SAU

**Keywords:** regional anesthesia, post-anesthesia care unit, pain, length of stay, general anesthesia

## Abstract

Background: The post-anesthesia care unit (PACU) plays a crucial role in providing specialized care to postoperative patients. However, a subset of these patients experiences complications that result in a prolonged stay of 90 minutes or more in the PACU. This not only impacts the patient's quality of life but also disrupts hospital workflow, as it might cause postoperative pain, nausea, or vomiting. It is essential to identify the factors contributing to this prolonged length of stay (LOS) and explore strategies for its prevention and management.

Methods: We conducted a retrospective cohort study of postoperative patients between 2020 and 2021. We included patients who had a prolonged stay, excluding cardiac patients, patients who had a planned prolonged stay, and patients waiting for an intensive care unit bed. We used a non-probability consecutive sampling technique. Data were obtained from the BestCare System, the hospital’s information system, using a data collection sheet.

Results: A total of 15,170 patients underwent surgical procedures during the study period, out of which only 181 (1.19%) experienced a prolonged PACU stay. Pain and altered mental status were strongly associated with a prolonged PACU stay (P = 0.035 and P = 0.0009, respectively). However, there was no significant association between overall comorbidities and prolonged LOS in the PACU, except for patients with asthma (P = 0.003). Different types and durations of surgeries did not significantly contribute to a prolonged PACU stay.

Conclusions: Our study found that among the various variables examined, asthma, pain, and altered mental status were significantly associated with a prolonged LOS in the PACU. These findings suggest that targeted interventions addressing these factors may help reduce the incidence of prolonged PACU stays and optimize patient outcomes.

## Introduction

The post-anesthesia care unit (PACU) was established worldwide in 1923, and it provides immediate care to all surgical patients undergoing any form of anesthesia [1]. In the PACU, patients receive specialized care, characterized by vigilant vital sign monitoring, assessment and treatment of postoperative pain, and ensuring a smooth recovery from anesthesia. However, despite the meticulous care provided, complications such as postoperative nausea and vomiting (PONV), loss of consciousness, respiratory depression, and changes in blood pressure can still occur, prolonging the patient's stay in the PACU [2-3]. Length of stay (LOS) in the PACU is defined as extended if it exceeds 90 minutes, which impinges on the well-being of patients and exerts a ripple effect on workflow, causing an overcrowding issue, limiting the bed availability and resources for new admission. Moreover, some postoperative complications are so severe that they not only prolong the PACU-LOS but also require recurrent assessments. Due to these considerations, it is crucial to consider the most common causes that extend patients' stay in the PACU, which are rooted in anesthetic, surgical, and patient-related factors [3]. Various factors may contribute to the prolonged stay of patients in the PACU. These factors include challenges related to pain management, unplanned postoperative ventilation requirements, and limitations stemming from the availability of specialized care beds [1]. Moreover, patient-related factors are beyond the control of medical staff. Specifically, advanced age (exceeding 60 years), gender (male), diagnosis of diabetes, and obesity exert a noteworthy effect. Moreover, patients presenting to the PACU with pain and PONV upon arrival tended to exhibit extended stays compared to their pain-free counterparts [2].

Researchers have found that several factors can trigger postoperative respiratory and cardiovascular events. In a study conducted at Unidade de Pronto Atendimento, a healthcare facility in Brazil, 140 of 206 (68%) patients experienced postoperative adverse events in the PACU. Thirty-six (25.7%) patients had cardiovascular-related events, such as hypotension or tachycardia, and 25 (17.8%) had respiratory problems [3]. The choice of anesthesia administered during surgical procedures has also emerged as a significant contributor to postoperative complications. General anesthesia is known for its manifold side effects, such as PONV and delayed awakening. However, PONV arises from multifaceted mechanisms, such as the effect of anesthesia on specific locations in the brainstem or alterations in the blood flow supply, ultimately eliciting the need for emesis [3]. Furthermore, geriatric, pediatric, and obese patients have demonstrated prolonged awakening following general anesthesia associated with extended PACU stays [4]. By contrast, patients who have undergone regional anesthesia typically report less pain and are more satisfied with their care. Nonetheless, regional anesthesia translates into superior postoperative outcomes in the form of fewer complications and a moderate LOS in the PACU. Studies have also shown that the type of procedure is related to the LOS in the PACU and postoperative pain; therefore, patients might need more analgesics in the PACU. General, thoracic, and orthopedic surgeries have been recognized as predisposing factors for prolonged PACU stays and heightened pain intensity [4]. Patients with plastic surgery remain healthy and have almost no risks [1]. Consequently, such patients, particularly those undergoing orthopedic surgery, require special care when being monitored for severe complications [1].

Therefore, this study was conducted to identify the underlying factors related to prolonged stay of patients within the PACU over the past two years at the King Abdulaziz Medical City (KAMC), Jeddah, and its correlation with other factors, such as type of surgery, type of anesthesia, and duration of surgery.

## Materials and methods

Study settings and subjects

We conducted a retrospective cohort study at the King Abdulaziz Medical City (KAMC), Western Province, a non-profit tertiary center that provides high-standard patient care to the western, northern, and southern parts of Saudi Arabia. The study participants of this study were PACU patients at the KAMC from January 2020 to December 2021. Inclusion criteria included all postoperative patients within the PACU who remained for 90 minutes and more. Exclusion criteria included patients with post-cardiac surgery, those planned for extended recovery, and those awaiting ICU admission. Initially, we anticipated 80-100 prolonged cases annually, but we were surprised to discover 897 prolonged cases, most of which were excluded due to logistical constraints. The modified Aldrete criteria was used to determine whether patients were eligible to be discharge to their hospital rooms or not.

Sampling technique

This study used a non-probability consecutive sampling technique that encompassed all patients who did not meet the exclusion criteria.

Data collection methods, instruments, and measurements

Data collection involved observing patient records through the BestCare and Anesthesia Centricity System and utilizing a data collection sheet to ensure accurate documentation of variables. Primary outcomes and grouping variables were categorized as surgery, anesthesia, patient-related, and others. Preoperative data included demographics, such as age, weight, height, infection, and comorbidities. The operative data included the type of surgery, anesthesia modality, surgery duration, and any operative complications. Postoperative variables, such as LOS in the PACU, pain score, respiration rate, oxygen saturation, spinal level, mental status, pain scores, blood pressure, and presence of nausea and or vomiting, were recorded.

Data management and analysis plan

Percentages were used to describe the categorical variables (sex, comorbidity, anesthesia used, PONV, and type of procedure performed). To assess the normality of the PACU LOS variable, we performed the Shapiro-Wilk test. The results indicated that the variable was not normally distributed and hence was presented as median and interquartile range (IQR). To evaluate the difference in the PACU LOS among demographic parameters, clinical characteristics, and postoperative variables, we used a Mann-Whitney test or Kruskal-Wallis test. If a statistical difference was detected, the comparison between groups was conducted using post-hoc tests (Mann-Whitney U test). These non-parametric tests are suitable for analyzing variables that are not normally distributed. Statistical significance was set at P < 0.05. Statistical analysis was performed using JMP Pro 14 software (JMP, Pro 14; SAS Institute Inc., 1989-2019).

Ethical approval

This study was conducted in accordance with the ethical standards as data were anonymized, and the study was approved by the King Abdullah International Medical Research Center (Institutional Review Board (IRB) approval ID: SP22J/018/02), which waived the requirement for informed consent.

## Results

We included 181 (1.19%) patients who had a prolonged stay in the PACU after exclusions according to our previously mentioned exclusion criteria. The lengths of these prolonged stays did not follow a normal distribution, with a median duration of 115 minutes. Among the patients, 43.09% were males and 56.91% were females. Table 1 presents the demographic characteristics and biometrics of the participants. The mean age of the patients was 40.9 years. Of 181 patients, 140 (78.45%) were adults. Data of the patients revealed that 68.33% of the patients had a high body mass index and 42.22% fell within the obesity range. However, there was no significant association between obesity and PACU LOS (P = 0.53). No significant association between PACU LOS and comorbidities, except in patients with asthma (P = 0.038). Among the different types of surgeries and their durations, there was no significant association with the PACU LOS (P = 0.55). In addition, although most patients received general anesthesia (N = 169, 93.37%) rather than regional anesthesia (N = 12, 6.63%), different types of anesthesia had no effect on prolonging the stay of patients in the PACU.

**Table 1:** Demographics, clinical characteristics, and length of stay within the PACU BMI = body mass index, ENT = ear, nose, and throat. * One patient with underweight BMI was excluded from the comparison. Statistical significance was set at P < 0.05. Statistical analysis was performed using JMP Pro 14 software (JMP, Pro 14; SAS Institute Inc., 1989-2019).

Variables	N (%)	Median (IQR)	P value
Sex:			0.57
Male	78 (43.09)	115 (42.5)	
Female	103 (56.91)	112.5 (54.25)	
Age category:			0.41
Pediatrics	39 (21.55)	120 (44.5)	
Adults	142 (78.45)	115 (53.75)	
BMI*:			0.53
Normal	57 (31.67)	115 (45)	
Overweight	47 (26.11)	115 (58.75)	
Obese	76 (42.22)	112.5 (46.25)	
Comorbidities:			0.53
Hypertension:	44 (24.3)	112.5 (43.25)	
Diabetes:	37 (20.44)	115 (31.5)	0.99
Dyslipidemia:	19 (10.59)	115 (40)	0.46
Anemia:	3 (1.65)	105 (25)	0.48
Cardiovascular disease:	11 (6.08)	125 (50)	0.39
Asthma:	8 (4.42)	157.5 (91.25)	0.038
Chronic kidney disease:	6 (3.31)	100 (21.25)	0.27
Obesity	76 (42.22)	115 (50)	0.62
Type of surgery:			0.55
Orthopedic	54 (29.84)	110 (45)	
General surgery	45 (24.86)	115 (50)	
Urology	13 (7.18)	110 (42.5)	
ENT	8 (4.42)	107.5 (54.25)	
Others	61 (33.70)	120 (60)	
Type of anesthesia:			0.19
General	169 (93.37)	115 (50)	
Regional	12 (6.63)	112.5 (36.5)	

Table 2 represents the postoperative-related factors. Several factors were investigated, such as oxygen saturation, spinal levels (numbness after anesthesia), mental status, respiration, blood pressure, the presence of nausea and/or vomiting, and pain, two of which were associated with a prolonged stay. Mental status showed a significant association (P = 0.046), where the LOS in the PACU was statistically significantly lower (median = 100) in patients with sleepy/comatose mental status compared to patients with drowsy/arousable mental status (median = 120, P = 0.01). Pain demonstrated strong evidence of a correlation with (P < 0.001).

**Table 2:** Postoperative complications and length of stay within the PACU * One patient is excluded. PACU = post-anesthesia care unit

Complications	N (%)	Median (IQR)	P value
Oxygen saturation:			0.15
Intubated	6 (3.31)	142.5 (72.5)	
Saturation 90% with support	127 (70.17)	115 (45)	
Saturation above 92%	48 (26.52)	105 (50)	
Spinal level:			0.13
No movement	8 (4.42)	102.5 (37.5)	
Mild movement	10 (5.52)	112.5 (65)	
Flex knee with resistance	15 (8.29)	109 (31)	
Free movement	148 (81.77)	115 (50)	
Mental status*:			0.0469
Sleepy, comatose	15 (8.33)	100 (25)	
Drowsy, arousable	106 (58.89)	120 (60)	
Awake, aware	59 (32.78)	110 (40)	
Respiration*:			0.65
Not breathing	4 (2.22)	115 (32.5)	
Shallow breathing	12 (6.67)	120 (70)	
Normal breathing	164 (91.11)	115 (50)	
BP:			0.2412
More than 40% of than baseline	5 (2.76)	100 (50)	
+/- 40% of baseline	21 (11.60)	135 (50)	
+/- 20% of baseline	155 (85.64)	115 (55)	
Presence of nausea and vomiting:			0.7857
Both	10 (5.53)	141.5 (58)	
None	86 (47.51)	110 (46.25)	
Unknown	85 (46.96)	115 (47.5)	
Pain:			0.0009
Yes	103 (56.91)	110 (40)	
No	78 (43.09)	125 (62.5)	

## Discussion

The results of this study provide insights into the most common factors that cause a prolonged LOS in patients in the PACU. Our investigation showed that age was not predictive of a prolonged hospital stay. However, 78.21% of the patients were adults, with the mean age of patients included to be 40.9 years. This finding might be explained by the fact that adults experience different pharmacodynamics that subject them more risks of anesthesia than the pediatric population [3]. For example, adults aged 40 years and older, which was the dominant demographic in our study, experienced a more observable decrease in first-pass metabolism [3]. This leads to delayed clearance of anesthesia from the systems, as well as a reduction in drug metabolism rates. Moreover, it might be caused by age-related changes in the sensitivity of receptors found in the central nervous system (CNS), which is the main target of most anesthetic drugs. Thus, this hypersensitivity poses a higher risk of postoperative complications in older patients, as the effects of anesthesia are prolonged [4].

Sex was not significantly correlated with a prolonged recovery (P > 0.05). Our results were contraindicated by Hartwan et al., in which sex was a significant factor in LOS in the PACU as female patients had better recovery quality and rates [5]. By contrast, Myles et al. found that male patients had a lower risk of postoperative complications than female patients [6].

Investigations during the data collection procedures revealed that 41.89% of the patients who had a longer length of hospital stay were obese and 26.25% were overweight. This result is consistent with well-established information on the association between obesity and poor functional and vital capacities, hypoventilation status, and various postoperative complications. Contrary to our clinical hypothesis, no significant correlation was observed between obesity and a prolonged LOS [7-9]. Similar studies have shown no statistically significant difference between patients with and without obesity and postoperative morbidity [10]. Moreover, a recent meta-analysis of 19 studies established that there was no significant difference in 30-day mortality or postoperative complications between patients with and without obesity [11]. We found that comorbidities, such as hypertension, diabetes, dyslipidemia, anemia, cardiovascular disease, and chronic kidney disease, were not significantly correlated with prolonged PACU LOS. This result contradicts the outcomes of Verbeeck et al. in which comorbidities, such as chronic kidney disease had an immense effect on the pharmacokinetics of anesthesia, resulting in delayed emergence in the PACU [12]. However, patients with asthma reported the longest LOS, higher pain scores, and an overall more complicated PACU stay. Moreover, the majority of patients with asthma in our study underwent elective rather than emergent surgeries, these patients received postoperative salbutamol and utilized nebulization masks. It is well established that patients with asthma have an increased risk of developing low blood oxygen levels due to post-anesthesia muscle spasms. Bronchospam is triggered by intubation during surgery. Moreover, a study conducted in 2018 by Numata et al. investigated postoperative pulmonary complications in asthmatic patients. They revealed that 7.0% of the patients with asthma included in the study experienced bronchospasm and required additional treatments [13]. Furthermore, the need for additional monitoring and treatment was proven during our investigation, as most patients with asthma typically rely on nebulizer masks for respiratory support until the effects of anesthesia subside [14].

In addition, we studied the PACU events that may have led to prolonged PACU LOS. We found that mental status alterations were the most common PACU complications among patients (P = 0.046), as 105 of the patients complained of drowsiness. This might be explained by patient-related factors that could affect fast recovery from anesthesia, such as age, or surgery-related factors, such as the medications administered during or before surgery [15]. We concluded that prolonged PACU LOS was related to high pain scores on admission to the PACU (P = 0.001), as 102 patients needed the administration of analgesics until their pain was relieved, and it was safe to discharge them. It was also found that most patients (93.29%) who had a prolonged stay in the past two consecutive years underwent general anesthesia, whereas only 6.7% had undergone regional anesthesia. A randomized controlled trial (RCT) has confirmed that the utilization of regional anesthesia correlates with shorter PACU LOS [16]. Furthermore, it has been clinically established that in comparison with general anesthesia, regional anesthesia provides excellent post-surgical pain management and pain scores, as well as lower postoperative cardiovascular, pulmonary, and gastrointestinal morbidity rates [17].

Our study showed no significant difference between the duration and type of surgeries with a prolonged stay. However, we found that most of the prolonged cases included in our study underwent orthopedic and general surgeries, which is consistent with a previous study performed in Saudi Arabia by Huda et al. [18] This might be because these surgeries have the highest incidence of intraoperative complications.

After extensive investigation, we found that most of our patients had unplanned prolonged stays in the PACU. This could be explained by the logistical factors that were dramatically affected by the COVID-19 crisis, in which medical wards were not available to admit most patients due to bed shortages, overwhelming nursing shifts, and delayed discharge orders by physicians. To our knowledge, our study is the first to investigate the relationship between the PACU LOS and surgical, anesthetic, and patient-related factors in Jeddah and the second in Saudi Arabia.

This study has several limitations. One limitation of this study is the heterogeneity of the study population. The main limitation we faced is poor data documentation by staff in the PACU due to the transition from paper documentation to digital documenting systems; however, the results of our study are nonetheless valid as they are supported by previous findings. In addition, the COVID-19 crisis affected the results of our study because some patients had to stay longer, owing to the unavailability of ICU beds. The sample included all PACU patients in KAMC who had a prolonged stay of more than 90 minutes; therefore, our study might not be representative of all patients.

## Conclusions

The results in this study showed that asthma, pain, and mental status were significantly associated with the prolongation of PACU duration. Factors like age, gender, BMI, type of anesthesia, type of surgery, and other complications, however, were not associated with a prolonged stay. Further studies with larger sample sizes are needed.
